# Versatility and outcomes of lateral arm free flap in head and neck reconstruction: a retrospective case series study of our experiences and innovations

**DOI:** 10.1016/j.bjorl.2023.101334

**Published:** 2023-09-19

**Authors:** Mailudan Ainiwaer, Lixiao Fan, Zheng Jiang, Chenyili Xiong, Fei Chen, Deying Gu, Jun Liu

**Affiliations:** Department of Otolaryngology, Head and Neck Surgery, West China Hospital, Sichuan University, Chengdu, China

**Keywords:** LAFF, Free flap, Head and neck reconstruction, MDASI, Psychosocial

## Abstract

**Objectives:**

The Lateral Arm Free Flap (LAFF) offers advantageous features like variable thickness and minimal hair growth. LAFF is primarily used in orofacial and pharyngeal reconstructions, with limited reports on tracheal/esophageal applications. Also, the psychological prognosis and quality of life in patients with LAFF reconstruction are lacking.

**Methods:**

This retrospective case series presents outcomes of LAFF reconstruction for head and neck surgical defects which included 19 patients. The disease type, location of reconstruction, TNM staging, tumor size, chemoradiation status, anxiety, depression, quality of life and detailed perioperative information of the LAFF reconstruction surgery were presented in a descriptive pattern. Subgroup analysis was conducted using Mann–Whitney *U* and Pearson *r* test.

**Results:**

All 19 patients (100%) had a viable flap after surgery with 5 (26.3%) of them developed flap-related complication. Patients with oropharyngeal reconstruction were more likely to have lower anxiety and depression score and higher quality of life than those with below-oropharyngeal reconstruction. However, no significant *p*-value was generated in any subgroup comparison or correlation test. And the application of LAFF in esophageal and tracheal reconstruction was proved to be successful.

**Conclusions:**

The lateral arm free flap exhibits remarkable versatility and multifunctionality, providing advantageous outcomes in head and neck reconstruction.

**Level of evidence:**

Level 4 according to OCEBM.

## Highlights

• Hypopharyngeal patients are more prone to psychosocial stress.
• The LAFF is a versatile and multi-purpose flap with high success rate.
• The LAFF can be used in esophageal and tracheal reconstruction.

## Introduction

Since its initial introduction in the early 1970s, free tissue transfer has gradually gained global recognition as a safe and durable procedure in head and neck surgery.^1^, ^2^, ^3^ Among the various free flaps developed, the Lateral Arm Free Flap (LAFF) was first pioneered by Song et al. in 1982 and further defined by Cormack and Katsaros in 1984.^4^, ^5^, ^6^ The LAFF relies on the Posterior Radial Collateral Artery (PRCA) located along the posterior aspect of the arm, offering advantageous features such as variable thickness, minimal hair growth, and the ability to facilitate primary closure without necessitating a skin graft, thereby reducing donor site morbidity.^7^, ^8^, ^9^

Notably, the LAFF has predominantly been employed in orofacial and pharyngeal reconstructions,^8^ with limited reports on its utilization in tracheal/esophageal reconstruction. Therefore, the aim of this study is to provide a comprehensive overview of our recent two-year experience employing the LAFF in head and neck reconstruction, while exploring the potential expansion of its application in this domain.

## Methods

This retrospective case series aims to present the outcomes of patients who underwent LAFF reconstruction for surgical defects in the head and neck region at West China Hospital between January 2021 and March 2023. The surgeries of 19 cases were performed individually by two surgeons, with the tumor resections and flap reconstruction being carried out by the same physician. The study population consisted of 19 patients, comprising 5 females (26.3%) and 14 males (73.7%), with a mean age of 59.1 years (ranging from 33 to 86). Among the included patients, 17 (89.5%) had a diagnosis of cancer, while the remaining two cases (Cases 7 and 18) were non-cancer patients. The basic demographics, cancer pathology type, flap statistics are summarized in Table 1, while Table 2 provides detailed postoperative treatment and complications of each patient.

**Table 1:** Basic demographic, disease and treatment data for 19 patients.

Case number	Gender	Age (year)	Pathology	TNM staging	Side of the LAFF	Recipient blood vessels	Estimated size of tumor (cm)
1	F	71	Papillary carcinoma, undifferentiated carcinoma of thyroid gland	T4bN1aM0	R	Not specified	7.5 × 8.0
2	F	77	Postauricular basal cell carcinoma	rT2N0M0	R	**A**: right superior thyroid.	2.8 × 1.8
						**V**: branch of right middle thyroid.	
3	M	58	Tongue squamous cell carcinoma (SCC)	T3N1M0	L	**A**: left superior thyroid.	3.0 × 4.1
						**V**: left external jugular.	
4	M	63	Hypopharyngeal poorly differentiated SCC	rT4bN0M0	L	**A**: facial.	3.5 × 3.6
						**V**: facial.	
5	M	65	Tonsillar SCC	pT3N1M0	L	**A**: left superior thyroid.	4.0 × 3.8
						**V**: 2 branches of left superior thyroid.	
6	F	60	Tracheal adenoid cystic carcinoma	T1N0M0	L	**A**: transverse cervical.	1.5 × 1.0
						**V**: transverse cervical.	
7	M	33	Caustic esophagitis, esophageal stricture	/	L	**A**: left superior thyroid.	8.0 × 5.0
						**V**: 3 branches of external jugular.	
8	M	55	Hypopharyngeal SCC	T3N2bM0	L	**A**: left superior thyroid.	N/A
						**V**: left external jugular (end-to-side).	
9	M	86	Hypopharyngeal SCC	T3N0M0	L	**A**: left superior thyroid.	N/A
						**V**: left external jugular.	
10	M	47	Tonsillar SCC	T2N0M0	L	**A**: right superior thyroid.	2.0 × 4.0
						**V**: right external jugular (end-to-side)	
11	M	58	Hypopharyngeal SCC	T3N2cM0	L	**A**: left superior thyroid.	2.3 × 1.8
						**V**: left facial, left superior thyroid.	
12	M	57	Tonsillar poorly differentiated SCC	pT3N2M0	L	**A**: right superior thyroid.	2.8 × 2.7
						**V**: branch of right facial and right external jugular.	
13	M	49	Maxillary mucoepidermoid carcinoma	T4aN0M0	L	**A**: left superior thyroid.	2.0 × 1.5
						**V**: branch of left facial and left external jugular.	
14	F	54	Tonsillar poorly differentiated SCC	T3N0M0	L	**A**: right superior thyroid.	3.1 × 2.7
						**V**: right facial.	
15	M	69	Laryngeal/hypopharyngeal SCC	T1aN0M0; T3N0M0	L	**A**: left superior thyroid.	N/A
						**V**: branch of left facial.	
16	M	62	Tracheal SCC	T1aN0M0	L	Not specified	N/A
17	M	55	Oropharyngeal SCC	T3N0M0	L	**A**: right superior thyroid.	0.7 × 3.0
						**V**: branch of right facial.	
18	F	45	Postoperative esophago-cutaneous fistula	/	L	**A**: right transverse cervical.	8.0 × 5.0
						**V**: 2 branches of external jugular.	
19	M	58	Hypopharyngeal SCC	T3N1M0	R	**A**: right superior thyroid.	5.0 × 6.0
						**V**: branch of left external jugular.

**Table 2:** Detailed information of the postoperative treatment and complication.

Case number	Radiotherapy or chemotherapy	Postoperative anticoagulant	Postoperative flap-related complication	Postoperative complications
1	**Postop**: chemoradiation (External medical record not available)	LMWH 6150AXaIU/day subcutaneous for 8-days	/	Hypoalbuminemia, hypokalemia
2	/	LMWH 6150AXaIU/day subcutaneous for 7-days	/	Mild facial nerve palsy
3	**Postop**: 70 Gy radiation	Enoxaparin 4000AXaIU/day for 1-day	/	Pneumonia
4	**Preop**: 60 Gy radiation	/	/	Mild wound infection
	**Postop**: paclitaxel + cisplatin + tislelizumab			
5	**Preop**: paclitaxel + cisplatin + tislelizumab	/	/	/
	**Postop**: 72 Gy radiation + paclitaxel + cisplatin			
6	/	/	/	Left facial pneumoderma
7	/	/	/	/
8	**Postop**: chemoradiation (External medical record not available)	LMWH 4100AXaIU/day subcutaneous for 6-days	/	/
9	**Postop**: chemoradiation (External medical record not available)	Enoxaparin 4000AXaIU/day for 3-days	/	Venous thrombosis
10	/	/	Hemorrhage	/
11	**Postop**: paclitaxel + cisplatin+70 Gy radiation	LMWH 4100AXaIU/day subcutaneous for 4-days	Hemorrhage	/
12	**Postop**: paclitaxel + cisplatin+70 Gy radiation	Enoxaparin 4000AXaIU/day for 3-days; rivaroxaban 15 mg bid	/	/
13	/	/	/	/
14	**Preop**: neoadjuvant tislelizumab	/	Mild purple discoloration of the flap on POD (postoperative day) 4	Pneumonia
15	**Postop**: chemoradiation (External medical record not available)	LMWH 4100AXaIU/day subcutaneous for 5-days	/	/
16	**Postop**: 60 Gy radiation	/	/	/
17	**Postop**: 70 Gy radiation	LMWH 4100AXaIU/day subcutaneous for 1-day	/	Swollen left arm, blisters
18	/	/	Pharyngeal fistula on POD3	/
19	**Postop**: 72 Gy radiation	LMWH 6150AXaIU/day subcutaneous for 4-days	Pharyngeal fistula on POD4	Hypoalbuminemia, pneumonia, pleural effusion

In this study, the LAFF were used primarily to reconstruct oropharyngeal defect (36.8%), hypopharyngeal defect (31.6%), tracheal defect (15.8%), esophagus (10.5%) and skin (5.3%). Among the cancer patients, 13 out of 17 patients (76.5%) had a stage ≥T3, 7 out of 17 patients (41.2%) had clinically confirmed lymph node metastasis. 12 out of 17 patients (70.6%) underwent postoperative radiotherapy and 8 out of 17 patients (47.1%) underwent chemotherapy.

Regarding LAFF surgery, the length of the flap (parallel to the long axis of arm) ranges between 5.5–10 cm, the width (perpendicular to the long axis of arm) ranges between 4.5–6 cm. Whether to use right arm or left arm based solely on the patient’s strong hand (weak hand was chosen as the donor side), with 3 patients (15.8%) underwent right LAFF reconstruction and 16 patients (84.2%) underwent left LAFF reconstruction. In terms of blood vessel anastomoses, superior thyroid artery was the most chosen artery for anastomoses, accounting for 73.7% (14 out of 19), second to it was transverse cervical artery (10.5%, 2 out of 19) and facial artery (5.3%, 1 out of 19). While the branches of external jugular vein accounted for 47.4% (9 out of 19), branches of facial vein accounted for 31.6% (6 out of 19), and thyroid vein branches accounted for 15.8% (3 out of 19).

During the postoperative care of patients, measures were taken to mitigate perioperative complications in high-risk individuals. Specifically, 10 out of the 19 patients (52.6%) deemed to have a high risk of venous thrombosis or cardiovascular emergencies were administered anticoagulants as a preventive measure. The evaluation was based on patient’s past medical history of circulatory diseases and at each surgeon’s discretion.

Furthermore, in order to assess the psychological well-being and symptom burden of the patients, a subset of 10 patients (52.6%) completed the Zung Self-rating Anxiety Scale (SAS)^10^ and Zung Self-rating Depression Scale (SDS)^11^ immediately prior to discharge. At the 1-month follow-up, 13 out of the 19 patients (68.4%) completed assessments using the SAS, SDS, and MDASI-H&N (MD Anderson Symptom Inventory for head and neck cancer)^12^ inventories at 1-month follow-up, detailed results from these assessments are provided in Table 3, Table 4.

**Table 3:** SAS and SDS inventories.

Case number	SAS score at discharge	SAS score at 1-month follow-up	SDS score at discharge	SDS score at 1-month follow-up
3	32	28	51 (mild)	54 (mild)
5	22	21	22	47
6	34	/	42	/
7	28	32	49	47
10	33	35	49	48
11	27	34	46	40
12	32	31	47	34
13	/	36	/	27
14	/	39	/	46
15	38	43	48	57 (mild)
16	/	39	/	47
17	34	36	42	42
18	/	47 (mild)	/	46
19	34	27	53 (mild)	55 (mild)

**Table 4:** The detailed scoring of MDASI-H&N inventory in LAFF patients.

	3	5	7	10	11	12	13	14	15	16	17	18	19
Pain	4	0	0	1	3	3	4	0	0	3	0	5	2
Fatigue	7	1	5	1	4	2	6	5	3	0	0	3	5
Nausea	2	0	5	0	0	0	3	2	0	0	0	4	5
Disturbed sleep	9	2	10	1	4	2	3	2	8	0	0	6	6
Distress	3	2	5	1	3	2	6	3	8	4	0	5	6
SOB	3	2	5	1	2	0	4	3	2	3	0	4	1
Memory	1	0	10	0	2	0	3	7	3	0	2	6	2
Appetite	1	0	10	0	0	0	2	8	5	3	0	5	2
Drowsy	2	0	0	0	0	0	4	8	3	0	0	2	2
Dry mouth	1	0	5	1	3	0	3	8	3	0	0	8	2
Sadness	1	0	0	0	3	0	4	2	8	5	0	9	2
Vomiting	1	0	0	0	0	0	3	0	0	0	0	4	6
Numbness or tingling	1	3	5	1	0	0	6	1	3	0	0	4	4
Mucus	4	1	5	1	4	0	4	3	8	0	8	5	6
Swallowing	6	2	8	1	10	0	5	4	8	0	0	4	6
Choking or coughing	8	0	8	1	0	4	3	2	0	0	0	4	6
Voice or speech	5	2	0	1	10	7	4	3	8	10	8	6	6
Skin pain	1	0	0	1	1	0	3	0	0	5	0	5	1
Constipation	9	2	3	0	0	0	3	0	0	0	0	5	1
Tasting food	1	0	0	1	0	0	4	10	0	0	0	5	1
Throat sore	8	0	3	1	4	0	4	1	0	0	0	6	1
Teeth or gum problem	1	0	0	1	2	0	4	0	0	0	0	6	1
General activity	1	0	0	0	0	2	3	0	7	3	0	7	1
Mood	3	1	0	0	4	2	0	1	9	3	0	7	1
Work	2	1	0	0	5	0	0	10	10	2	3	5	1
Relation	2	0	0	0	3	0	0	2	8	10	3	5	1
Walking	4	1	0	1	0	2	0	3	3	0	0	6	1
Enjoyment of life	3	1	0	0	4	2	0	3	9	3	3	4	1
Total score	94	21	87	16	71	28	88	91	116	54	27	145	80

Statistical analysis was performed using R 4.2.3, mean scores were presented without reporting standard deviation due to too small sample size. Subgroup analysis was conducted using the Mann–Whitney *U* test and Pearson *r* correlation test. A significance level of *p* < 0.05 was considered statistically significant in all analyses.

## Results

### Complications

In this case series study, a noteworthy finding was observed: all 19 flaps achieved complete survival without any instances of partial or complete flap loss, indicating a remarkable 100% flap survival rate. Nevertheless, a subset of five patients (26.3%) encountered flap-related complications during the postoperative course.

Notably, in Case 14, mild purple discoloration of the flap was discovered via laryngoscopy on postoperative day 4, indicating a potential compromise in venous return. Case 19 developed a mild pharyngeal fistula, which fortunately did not necessitate any surgical intervention. However, Case 18 experienced a persistent pharyngeal fistula, resulting in continuous drainage for a duration of five months following the initial procedure. Subsequently, the patient required re-operation to address the persistent fistula. Cases 10 and 11 experienced hemorrhages at the surgical site, necessitating re-operation to achieve hemostasis. Both of them had an active hemorrhage from the flap anastomosis site, Case 10 had a hematoma in the surgical field which was removed by open surgery. While Case 11 had a mild hemoptysis, which was successfully managed by open surgical exploration and electrocoagulation.

Case 1 experienced hypoalbuminemia and hypokalemia, which were successfully resolved through enhanced nutrition management. Similarly, Case 2 developed mild facial nerve palsy, attributed to increased tension on the facial nerve during surgery, with gradual resolution observed over a two-month period. Pneumonia occurred in Cases 3 and 14, effectively managed with the administration of appropriate antibiotics. Case 4 presented with wound infection, which was successfully treated using antibiotic therapy. Mild pneumoderma, potentially resulting from tracheostomy, was observed in Case 6. Superficial venous thrombosis manifested in Case 9, an elderly patient with poor overall physical condition, and was effectively managed with enoxaparin. Case 17 exhibited swelling of the arm on postoperative day 2, along with bruising around the surgical wound and tension blisters on the forearm. The symptoms subsided following discontinuation of LMWH use. Case 19 developed hypoalbuminemia, pneumonia, and pleural effusion, all effectively managed through enhanced nutritional support and appropriate antibiotic therapy.

### Anxiety and depression

At discharge, 10 patients completed the Self-rating Anxiety Scale (SAS) and Self-rating Depression Scale (SDS) inventories. Among these patients, only Cases 3 and 19 exhibited mild depression. At the 1-month follow-up, 13 patients completed the SAS and SDS inventories. Case 18 demonstrated mild anxiety, while Case 15 exhibited mild depression. Importantly, the mild depression observed in Cases 3 and 19 persisted since discharge.

To further investigate the psychological impact of different reconstruction types, patients were manually regrouped into two categories: oropharyngeal reconstruction and below-oropharyngeal reconstruction groups. The below-oropharyngeal reconstruction group tended to have higher mean SAS and SDS scores compared to the oropharyngeal reconstruction group at discharge (32.20 > 30.60; 47.60 > 42.20), with this distinction becoming more prominent at the 1-month follow-up (37.00 > 32.29; 48.66 > 42.57).

Surprisingly, when examining the association between perioperative complications and psychological well-being, patients without any complications tended to have lower mean SAS scores (30.60 < 31.67) but higher mean SDS scores (48.00 > 43.60) at discharge. However, this trend completely reversed at the 1-month follow-up, where patients without complications tended to have higher SAS scores (36.20 > 33.38) but lower SDS scores (42.40 < 47.25). Notably, none of the cross-comparisons reached statistical significance in the Mann–Whitney *U* test.

### Quality of life assessment

A total of 13 patients completed the MDASI-H&N inventory, revealing a mean score of 70.61 (range: 21–145), indicative of a diverse range of symptom experiences among the patient cohort. Interestingly, the mean score for the oropharyngeal group was significantly lower than that of the below-oropharyngeal group (52.14 < 92.17), aligning with our clinical observations that patients undergoing hypopharyngeal, tracheal, or esophageal reconstruction tend to exhibit poorer postoperative quality of life. Notably, within the 28 items of the inventory, speech and swallowing difficulties emerged as the most prevalent issues in the below-oropharyngeal group, with mean scores of 6.67 and 6.00, respectively. These scores were considerably higher compared to the oropharyngeal group (4.29 and 2.57, respectively). Additionally, disturbed sleep and distress were also notable concerns affecting the quality of life in the below-oropharyngeal group.

Surprisingly, patients without perioperative complications demonstrated higher mean scores than those with complications (74.60 > 68.13), with speech function identified as the most affected domain in both groups (mean score of 5.80 in the no complication group and 5.13 in the complication group). However, it is important to note that none of the p-values obtained from the subgroup comparisons reached statistical significance, likely due to the limited sample size.

### Association between size of flap and postoperative quality of life

Flap size was calculated by multiplying the length and width measurements, and the Pearson *r* test was conducted to assess the associations between flap size and different outcome measures. The analysis generated five correlation coefficients (*r*-values) to examine the relationships between flap size and postoperative outcomes: 1) MDASI score (*r* = −0.027, *p* = 0.93), 2) SAS at 1-month follow-up (*r* = 0.013, *p* = 0.97), 3) SDS at 1-month follow-up (*r* = −0.194, *p* = 0.53), 4) SAS at discharge (*r* = 0.125, *p* = 0.73), and 5) SDS at discharge (*r* = 0.048, *p* = 0.90).

Notably, none of the correlation coefficients yielded statistically significant *p*-values (>0.05). These findings suggest that there are no significant correlations between flap size and postoperative quality of life outcomes in this study.

### Case reports of extended use of LAFF

In this study, we explore the novel utilization of LAFF in esophageal and tracheal reconstruction, aiming to evaluate the functional outcomes and associated complications.

**Figure 1:**
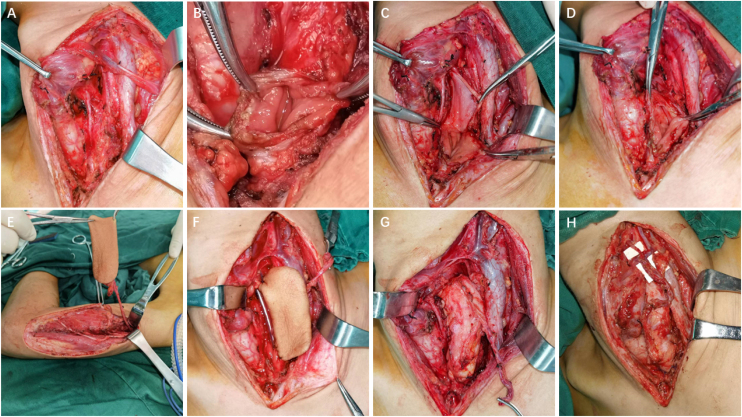
Detailed surgical processes for esophageal reconstruction in Case 11.

Case 7 involved a complex caustic esophagitis scenario, characterized by persistent stricture despite multiple unsuccessful treatment attempts, including balloon dilation and gastric pull-up surgery. The patient underwent excision of the scar tissue in the esophagus, followed by LAFF reconstruction to expand the lumen diameter, resulting in an optimal functional outcome. A lateral neck incision was performed, and the throat was pulled to the right to fully expose the cervical esophagus (Fig. 1A). A vertical incision on the esophagus was made at the stricture (Fig. 1B and C). The scar tissue along with the esophageal wall was excised (Fig. 1D). An 8 × 5 cm LAFF was harvested (Fig. 1E) with the pedicle measuring around 10 cm long. The LAFF was sewed to the vertical incision of the esophagus using 4‒0 Vicryl, a Nasogastric Tube (NGT) was inserted (Fig. 1F and G). The flap artery was anastomosed to the left superior thyroid artery using 8‒0 prolene suture and the three veins was anastomosed to three branches of the left jugular vein using 8‒0 prolene (Fig. 1H). The recovery was uneventful, his NGT was removed on the one-month follow-up and the patient had no problem eating or drinking, a laryngoscopy on three-month follow-up showed viable flap with hair growth, no obvious stricture of cervical esophagus was observed.

In contrast, Case 18 with similar esophageal reconstruction presented a less favorable outcome. Although the flap itself survived, the patient developed a persistent fistula extending from the suprasternal fossa to the cervical esophagus, leading to continuous drainage for a duration of 5-months, despite diligent wound management. Subsequently, re-operation was performed to excise the flap and a portion of the cervical esophagus. Reconstruction of the cervical esophagus was achieved using a jejunal flap.

**Figure 2:**
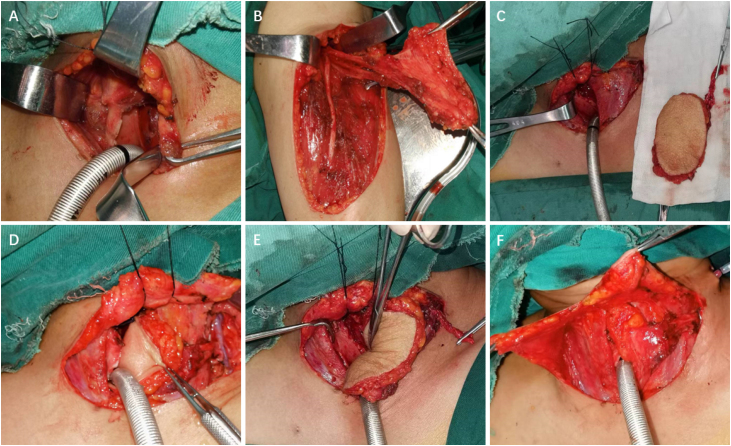
Detailed surgical process for tracheal reconstruction in Case 6.

**Figure 3:**
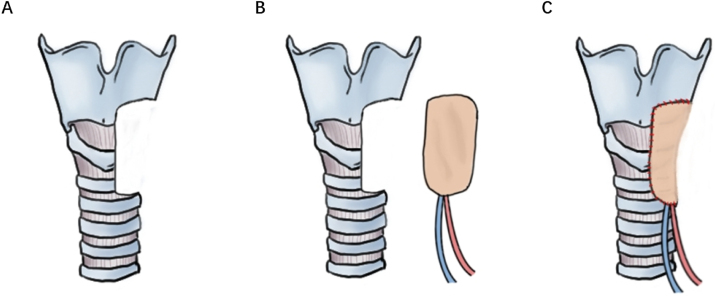
Schematic LAFF for tracheal defect reconstruction in Case 6.

For Cases 6 and 16, LAFF was employed to reconstruct non-circumferential defects of the trachea and a portion of the larynx. Taking Case 6 as an example, the patient had a tracheal adenoid cystic carcinoma extending from cricoid cartilage to 5th tracheal ring. A U-shaped incision was made, and the larynx and trachea were exposed. A part of larynx left portion of the trachea as well as left thyroid gland were excised (Figs. 2A, 3A), and a left side elective neck dissection was conducted. Subsequently, an 8 × 5 cm LAFF was harvested (Fig. 2B and C) and was anastomosed to the defect (Figs. 2D and E, 3B), and transverse cervical artery and vein were used for vascular anastomosis (Figs. 2E, 3C). Skin closure was done by 4‒0 Vicryl (Fig. 2F). After surgery, the patient experienced a mild facial pneumoderma, which resolved spontaneously without intervention. Both Cases 6 and 16 underwent successful decannulation and achieved favorable functional outcome.

## Discussion

Complex head and neck defects following surgery can significantly impact patients, affecting both functional abilities and aesthetic appearance. Successful restoration of these defects plays a crucial role in alleviating symptoms and improving the overall quality of life for patients. While Lateral Arm Free Flap (LAFF) is not as commonly utilized in head and neck reconstruction, it offers distinct advantages over other types of free flaps. One notable advantage of LAFF lies in its ability to provide a favorable skin color match and sufficient fat volume for head and neck reconstruction, without requiring excessive tissue volume. Unlike other flaps, LAFF offers excellent aesthetic outcomes, enabling surgeons to achieve a more natural appearance.^8^ Additionally, LAFF allows for primary closure without the need for skin grafting, as long as the flap width remains lower than 6–8 cm.^13^

When compared to free flaps harvested from lower limbs, such as the anterolateral thigh flap or posterior tibial artery perforator flap, LAFF demonstrates clear superiority. Harvesting a flap from the lateral arm is a more convenient process, as it eliminates the need to relocate personnel and equipment from the lower limbs. This not only reduces the risk of contaminating the surgical field but also enhances procedural efficiency. Furthermore, LAFF patients generally experience faster postoperative recovery, enabling earlier ambulation compared to those undergoing lower limb flap reconstruction. This reduced immobility contributes to a decreased risk of complications such as deep venous thrombosis and aspiration pneumonia.^14^ In comparison to the widely used radial forearm free flap in head and neck reconstruction, LAFF offers additional advantages. Notably, LAFF eliminates the requirement for skin grafting and provides a higher tissue volume, making it an attractive option for surgeons and patients alike.^15^

While the LAFF offers several advantages in head and neck reconstruction, certain limitations have hindered its widespread adoption in reconstructive surgery. The most notable drawback is its short vascular pedicle and thin blood vessel diameter.^16^ To address this issue, Wang et al. proposed a modification to the technique by cutting off the blood vessel at the level of the deep brachial artery, resulting in a longer and wider anastomosing artery.^17^ This modification, known as modified LAFF (mLAFF), has shown promising outcomes with minimal donor site complications, thereby enhancing the potential of LAFF in head and neck reconstruction.^17^

In our case series study, we observed that all patients retained flap viability immediately after surgery, and no flap loss were reported. Although one patient experienced a persistent fistula that required reoperation after 5-months, the overall flap survival rate in our study aligns with a pooled analysis from a previous review by Kang et al. (96.3%).^8^ This finding suggests that the small vessel caliber and short pedicle associated with LAFF do not necessarily compromise flap viability.

In terms of postoperative complications, the postoperative hemorrhage was the most common complication which happened in two patients. The anticoagulation therapy is usually considered as the contributing factor to the postoperative complication. However, several studies have indicated that the specific anticoagulant drug types and treatment protocols appear to have no significant correlation with flap survival and may even pose a risk of bleeding.^18^, ^19^, ^20^, ^21^, ^22^ Research has demonstrated that flap outcomes are more closely associated with factors such as radiation therapy history, reconstruction site, ischemic time, and anastomotic revision.^23^ A very recent retrospective study focusing on anastomotic revisions of flaps has shown that anticoagulation treatment regimens and the presence of congestion in flaps are not significantly correlated with flap failure.^24^ In clinical practice, whether to use anticoagulants after flap surgery is largely based on the patient’s baseline condition and at each surgeon’s discretion. In our study, most of the patients without anticoagulation therapy were doctor F.C.’s patient, meanwhile, doctor J.L. is relatively more prone to use anticoagulation therapy. Among the patients who underwent postoperative anticoagulation therapy, two patients were put on anticoagulation therapy due to accompanying disease. Patient 3 had a history of coronary stent placement and had discontinued anticoagulation therapy for one week prior to the surgery; Patient 9 exhibited left cephalic vein thrombosis on the 4th day after surgery. Two patients experienced blood vessel-related complications, Patient 11 experienced oral and nasal bleeding 4 h after surgery, necessitating surgical field exploration and hemostasis procedure; Patient 17 exhibited significant swelling in the left upper arm and bruising at the donor site on the 2nd day after surgery. Anticoagulation therapy was then discontinued, and the donor site was opened for decompression. Among all patients without anticoagulation treatment, two patients developed blood vessel-related complication, Patient 10 a hematoma was found at the anastomosis site, which was removed, and the draining tube was repositioned for a better drainage; for Patient 14, congestion of the skin flap occurred on the 3rd day after surgery, accompanied by fever, abnormal blood counts, and other signs of infection. Moreover, patients who did not receive anticoagulation treatment did not experience flap vascular anastomotic thrombosis or venous thrombosis events.

Due to the limited sample size, we did not observe a significant association between anticoagulation treatment and flap outcomes. Postoperative anticoagulation treatment protocols may vary based on the operating physician. Doctor J.L. tends to routinely administer postoperative anticoagulation treatment with a relatively fixed regimen. Conversely, Doctor F.C.’s approach is different; routine anticoagulation treatment postoperatively is not employed unless there is evidence of underlying cardiovascular conditions in patients or evidence of venous thrombus. Regarding the limited evidence at present, assessing the patient’s thrombotic risk preoperatively and evaluating their overall condition can provide valuable guidance for postoperative anticoagulation treatment. The anticoagulation strategy should be customized on an individual basis.

For postoperative quality of life assessment, we chose MDASI-H&N instead of EORTC QoL H&N35 because we aimed to focus more on capturing the symptom burden and impact on daily functioning in head and neck surgery patients. The MDASI inventory includes a comprehensive assessment of multiple symptoms and their effects, allowing for a detailed evaluation of symptom severity and interference with daily life.^12^ However, the MDASI-H&N may not cover broader quality of life domains outside of symptomatology, in future studies, we’ll try to combine these two inventories together to generate a more comprehensive postoperative quality of life picture of our patients.

Based on the location of reconstruction (oropharyngeal vs. below oropharyngeal) and the presence or absence of complications, we further classified patients into four subgroups, aiming to evaluate the impact on postoperative anxiety, depression, and quality of life. As expected, the below-oropharyngeal reconstruction group exhibited higher Scores in Anxiety (SAS), Depression (SDS), and the MDASI-H&N inventories, given the typically more extensive surgical procedures and the prevalence of speech and swallowing difficulties postoperatively.^25^ However, no significant results were obtained in other subgroup and correlation analyses, potentially due to the limited sample size. In addition, confounders like tumor staging, oral feeding and tracheostomy can significantly affect the correlation. Thus, a larger population and a more robust statistical analysis are required in the future prospective study, which is ongoing at our department, which includes all types of flaps in head and neck reconstruction.

We have successfully extended the application of the LAFF to include esophageal and tracheal reconstruction, a previously unexplored area. Particularly, for non-circumferential defects, LAFF serves as a favorable alternative in reconstructing the esophagus and trachea. Its utility is not limited to defect reconstruction following tumor resection in cancer patients but also extends to addressing esophageal strictures and complex fistulas.

## Conclusions

In summary, the lateral arm free flap exhibits remarkable versatility and multifunctionality, providing advantageous outcomes in head and neck reconstruction. It ensures a favorable skin color match and ample fat volume, which are crucial for achieving optimal aesthetic results. However, it is important to acknowledge the limitations associated with the flap’s short vascular pedicle and thin vessel diameter, which can be mitigated through the implementation of the mLAFF technique. Further research, incorporating larger sample sizes, is warranted to comprehensively evaluate and compare LAFF with other free flap options in various clinical scenarios.

## Funding

This research has been funded by the Technology Department of Sichuan Province. The project is named the “Key Research and Development Program of Sichuan Province”. Grant number: 2022YFS0065.

## Institutional review board statement

This research is approved by the Institutional Review Board of the West China Hospital, reference number: 2019–510.

## Informed consent statement

Written informed consent for publication of this paper was obtained from The Institutional Review Board of the West China Hospital as well as directly from the patient.

## Data availability statement

All the available data is included in the article.

## Conflicts of interest

The authors declare no conflicts of interest.

## References

[1] Steel B.J., Cope M.R. (2015). A brief history of vascularized free flaps in the oral and maxillofacial region. J Oral Maxillofac Surg.

[2] Liu J., Ren J., Lv D., Wang J., Deng D., Li L. (2019). Simultaneous tracheal and esophageal reconstruction for thyroid cancer involving trachea and esophagus using a free bipaddled posterior tibial artery perforator flap. Head Neck.

[3] Deng D., Xu F., Liu J., Li B., Li L., Liu J. (2020). Clinical application of pedicled thoracoacromial artery perforator flaps for tracheal reconstruction. BMC Surg.

[4] Song R., Song Y., Yu Y., Song Y. (1982). The upper arm free flap. Clin Plast Surg.

[5] Cormack G.C., Lamberty B.G. (1984). Fasciocutaneous vessels. Their distribution on the trunk and limbs, and their clinical application in tissue transfer. Anat Clin.

[6] Katsaros J., Schusterman M., Beppu M., Banis J.C., Acland R.D. (1984). The lateral upper arm flap: anatomy and clinical applications. Ann Plast Surg.

[7] Deleyiannis F.W.B., Gastman B.R., Russavage J.M., Myers E.N., Carrau R.L., Eibling D.E. (2008). Operative otolaryngology: head and neck surgery (Second Edition).

[8] Kang S.Y., Eskander A., Patel K., Teknos T.N., Old M.O. (2018). The unique and valuable soft tissue free flap in head and neck reconstruction: lateral arm. Oral Oncol.

[9] Marques Faria J.C., Rodrigues M.L., Scopel G.P., Kowalski L.P., Ferreira M.C. (2008). The versatility of the free lateral arm flap in head and neck soft tissue reconstruction: clinical experience of 210 cases. J Plast Reconstr Aesthet Surg.

[10] Zung W.W. (1971). A rating instrument for anxiety disorders. Psychosomatics.

[11] Zung W.W. (1965). A self-rating depression scale. Arch Gen Psychiatry.

[12] Rosenthal D.I., Mendoza T.R., Chambers M.S., Burkett V.S., Garden A.S., Hessell A.C. (2008). The M.D. Anderson symptom inventory-head and neck module, a patient-reported outcome instrument, accurately predicts the severity of radiation-induced mucositis. Int J Radiat Oncol Biol Phys.

[13] Amin J.D., Amin N., Hatten K.M. (2020). The lateral arm free flap for head and neck reconstruction. Curr Opin Otolaryngol Head Neck Surg.

[14] Talec P., Gaujoux S., Samama C.M. (2016). Early ambulation and prevention of post-operative thrombo-embolic risk. J Visc Surg.

[15] Médard de Chardon V., Balaguer T., Chignon-Sicard B., Riah Y., Ihrai T., Dannan E. (2009). The radial forearm free flap: a review of microsurgical options. J Plast Reconstr Aesthet Surg.

[16] Yang X.-D., Zhao S.-F., Zhang Q., Wang Y.-X., Li W., Hong X.-W. (2016). Use of modified lateral upper arm free flap for reconstruction of soft tissue defect after resection of oral cancer. Head Face Med.

[17] Wang W.-M., Sun L., Yang S.-S., Hu S.-J., Zuo Y.-J., Min A.-J. (2022). Comparison between modified lateral arm free flap and traditional lateral arm free flap for the reconstruction of oral and maxillofacial soft tissue defects. Front Oncol.

[18] Cevik J., Middleton R., Ramakrishnan A., Cabalag M. (2021). Rationalizing post-operative prophylactic anticoagulation in reconstructive head and neck cancer patients: a review. ANZ J Surg.

[19] Barton B.M., Riley C.A., Fitzpatrick J.C., Hasney C.P., Moore B.A., McCoul E.D. (2018). Postoperative anticoagulation after free flap reconstruction for head and neck cancer: a systematic review. Laryngoscope.

[20] Liu J., Shi Q., Yang S., Liu B., Guo B., Xu J. (2018). Does postoperative anticoagulation therapy lead to a higher success rate for microvascular free-tissue transfer in the head and neck? A systematic review and meta-analysis. J Reconstr Microsurg.

[21] Swartz J.E., Aarts M.C.J., Swart K.M.A., Disa J.J., Gerressen M., Kuo Y.-R. (2015). The value of postoperative anticoagulants to improve flap survival in the free radial forearm flap: a systematic review and retrospective multicentre analysis. Clin Otolaryngol.

[22] Cannady S.B., Hatten K., Wax M.K. (2016). Postoperative controversies in the management of free flap surgery in the head and neck. Facial Plast Surg Clin North Am.

[23] Zhou W., Zhang W.-B., Yu Y., Wang Y., Mao C., Guo C.-B. (2017). Risk factors for free flap failure: a retrospective analysis of 881 free flaps for head and neck defect reconstruction. Int J Oral Maxillofac Surg.

[24] Bishop J.L., Vasudev M., Garcia N., Heslop G., Pham T.T., Hicks M.D. (2023). Effect of perioperative antithrombotics on head and neck microvascular free flap survival after anastomotic revision. Otolaryngol Head Neck Surg.

[25] Mahalingam S., Srinivasan R., Spielmann P. (2016). Quality-of-life and functional outcomes following pharyngolaryngectomy: a systematic review of literature. Clin Otolaryngol.

